# Associations between the Gut Microbiota, Immune Reconstitution, and Outcomes of Allogeneic Hematopoietic Stem Cell Transplantation

**DOI:** 10.20900/immunometab20210004

**Published:** 2021-01-12

**Authors:** Salvatore Fiorenza, Cameron J. Turtle

**Affiliations:** 1 Clinical Research Division, Fred Hutchinson Cancer Research Center, 1100 Fairview Ave N, Seattle, WA 98109, USA; 2 Department of Medicine, University of Washington, 1959 NE Pacific St, Seattle, WA 98195, USA

**Keywords:** microbiome, allogeneic, transplant, metagenomics, immune reconstitution

## Abstract

Immune reconstitution following allogeneic hematopoietic stem cell transplantation (allo-HSCT) sets the stage for the goal of a successful transplant—the prevention of disease relapse without graft versus host disease (GVHD) and opportunistic infection. In both epidemiologic studies and in controlled animal studies, it is known that the gut microbiome (GM) can profoundly influence normal innate and adaptive immune development and can be altered by microbial transfer and antibiotics. Following allo-HSCT the GM has been shown to influence clinical outcomes but published associations between the GM and immune reconstitution post-allo-HSCT are lacking. In this viewpoint we propose that the extensive knowledge garnered from studying normal immune development can serve as a framework for studying immune development post-allo-HSCT. We summarize existing studies addressing the effect of the GM on immune ontogeny and draw associations with immune reconstitution and the GM post-allo-HSCT.

## INTRODUCTION

Allogeneic hematopoietic stem cell transplantation (allo-HSCT) is a curable modality for many hematologic malignancies and bone marrow disorders [1]. Following conditioning chemotherapy, donor HSCs and co-infused donor immune cells establish new innate and adaptive immune systems that are anticipated to protect the host from infection and relapse—the latter referred to as a graft versus malignancy (GvM) effect [2,3]. Immune reconstitution, however, carries the risk of graft versus host disease (GvHD)—a multisystem acute and/or chronic immune-mediated disease, characterized by donor cell reactivity to host tissues [4]. Immunosuppressive therapies (IST) have improved GvHD-related mortality [1]; however, the ability to consistently eliminate GvHD without impacting GvM and infection risk is lacking. The gut microbiome (GM) has been shown to fine-tune elements of the developing immune system in infancy, resulting in lasting impacts on immunity throughout life [5]. Emerging evidence is now revealing that the GM can also impact immune development post-allo-HSCT in murine models and in patients [6–8]. We review the role the GM plays in shaping neonatal immune development, and how this can serve as a framework that will guide further investigations into how GM signals alter immunity post-allo-HSCT.

## THE IMPORTANCE OF POST-ALLO-HSCT IMMUNE RECONSTITUTION KINETICS

**Figure 1.**
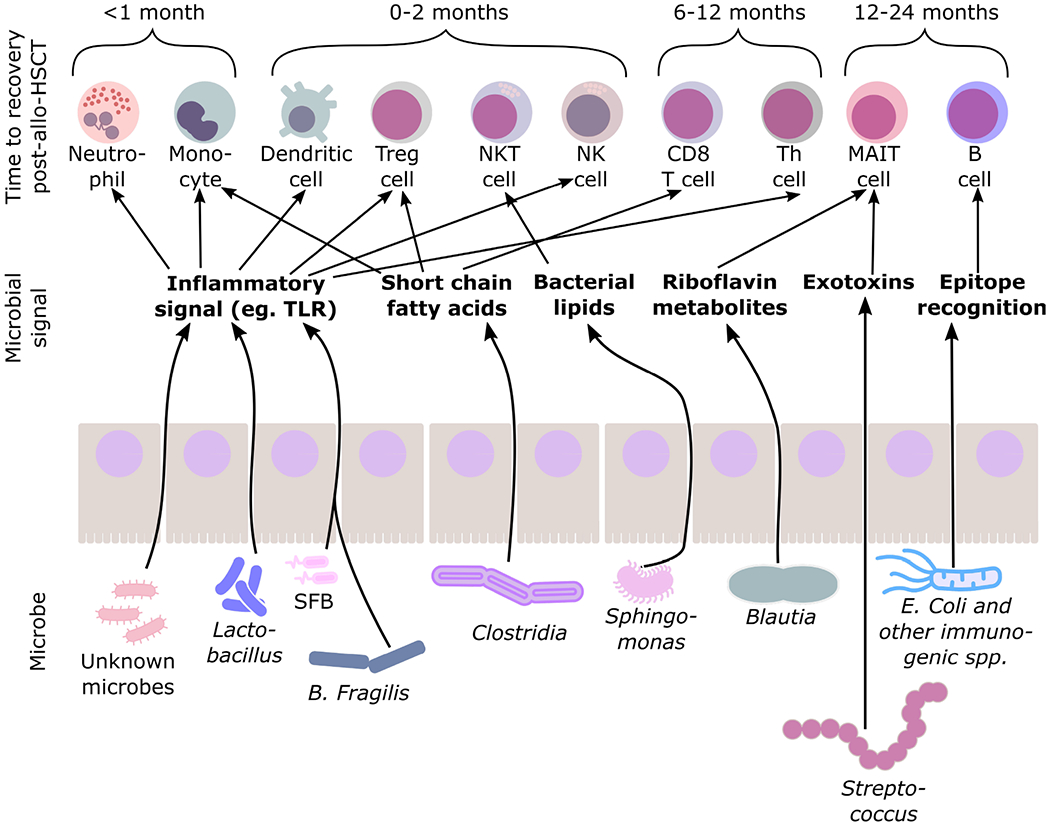
Time to recovery of immune cell subsets following allogeneic hematopoietic stem cell transplantation (allo-HSCT) and representative associations between components of the gut microbiome and selected immune subsets in normal development. Abbreviations: NK, natural killer; Th, T helper cells; Treg, Regulatory T cell; MAIT, mucosal-associated invariant T cell; SFB, segmented filamentous bacteria. References as per Table 2.

Immune reconstitution post-allo-HSCT occurs over approximately two years, encompassing the recovery of both effector and regulatory immune cell subsets (Figure 1). For an in-depth discussion of the impacts of donor and host variables on the kinetics of immune recovery the reader is referred to excellent recent reviews [3,9]. Studies assessing the impact of the magnitude and speed of immune recovery on allo-HSCT outcomes are mostly retrospective cohort analyses from single centers and are thus subject to variations in clinical practice, such as conditioning regimens, HSC dose, GVHD prophylaxis, ex vivo HSC manipulation [3,10,11]. Nevertheless, interesting insights into recovery of adaptive and innate immunity have been obtained and are summarized in Table 1.

Enhanced neutrophil recovery provides innate immune defense against bacterial and fungal infections and can shorten hospital stay, yet has been associated with a risk of potentiating GvHD [12]. Likewise, increased natural killer (NK) cell recovery at days 28 and 56 decreases the risk of relapse, but may come at the expense of increased risk of GVHD [13,14]. Although early monocyte recovery is associated with decreased risk of relapse and improved overall survival, [15–17] early recovery of immune regulatory myeloid derived-suppressor cells at day 14 increases the risk of both relapse and paradoxically severe acute GVHD [18].

Within the adaptive immune system, the extent and timing of recovery of distinct T cell subsets has important clinical implications. Early CD8^+^ T cell recovery is associated with decreased relapse risk and viral infection, but increased risk of acute GVHD [19–22]. Early reconstitution of CD45RA^−^ CD62L^−^ regulatory T cells (Tregs) at 3 months and long-lived, CD45RA^+^ CD62L^+^ Tregs at 6 months is associated with decreased incidence of chronic GVHD [23,24]. Rapid reconstitution of non-conventional T cells may influence the risk of post-transplant opportunistic infections (in the case of γδ T cells), GVHD (NKT and mucosal-associated invariant T, MAIT cells), and relapse (γδ and NKT cells) [25–28]. Early B cell recovery is associated with superior anti-viral, anti-bacterial immunity and response to vaccination and decreased non-relapse mortality [14,29]. Thus, the kinetics of reconstitution of numerous distinct immune subsets contribute to the outcomes of allo-HSCT.

## THE GM AND IMMUNE DEVELOPMENT

Since the association between GM composition in childhood and autoimmune disease in later life was established in the 1990s [35,36] there have been intense investigations into the mechanisms by which the GM composition impacts development of adaptive and innate immune cell stimulation and development in both mice and human cells in culture (Figure 1 and Table 2). By comparing mice raised in completely germ free (GF) environments to mice exposed to known microbes (gnotobiotic), or against mice harboring an array of normal microbes but lacking in certain pathogens (specific-pathogen-free, SPF) with or without antibiotic exposure, multiple studies have demonstrated the impact of the GM on specific components of immune development and subsequent immunity [37]. For example, absolute T cell and B cell counts are similar between SPF and GF mice, but CD4^+^ T-helper (Th) cell numbers are lower and Th2-biased cells higher in GF mice—a phenotype that can be rescued in GF mice by oral administration of bacterial polysaccharide (PSA) from the gut commensal *Bacteroides fragilis* [38]. PSA signals via toll-like receptor 2 (TLR2) to thymic-migrating dendritic cells (DCs), which can regulate PLZF-dependent transcriptional programs in developing thymic T cells, resulting in protection against autoimmune colitis and fatal inflammation from herpes viral encephalitis [38–40]. Segmented filamentous bacteria adhere to epithelial cells of the gut and in doing so create an inflammatory environment that supports the development Th17 cells that in turn cause ulcerative colitis in mouse models, which can be mitigated by the transplantation of Treg activating GM species [41,42]. Other pro-inflammatory bacterial components, such as exotoxins from Group A Streptococcus, have been shown to augment Treg proliferation in human cells in vitro and function through a monocyte dependent-mechanism thereby protecting against autoimmune disease in mouse models; however, it remains to be seen if this phenomenon is related to an initial inflammatory response or represents a distinct developmental program [43].

B cell development, and the immunoglobulin A (IgA) repertoire in particular, is shaped by sampling and presentation of GM antigens by subepithelial mesenchymal cells and dendritic cells within mucosal-associated lymphoid tissue [56]. Unlike systemic bacterial immunization which drives IgG-mediated responses with only low bacterial numbers, high bacterial loads are required for the development of antigen-specific IgA-mediated immunity [54,55]. These distinct thresholds and requirements for constant sampling and presentation allow directed polyclonal IgA responses in the face of antigenic diversity within the GM, whilst somatic hypermutation within the B cell compartment allows diversification of the IgA-secreting repertoire [55,57,58].

Metabolic byproducts of the gut microbiome can influence development of distinct T cell subsets. MAIT cells express an invariant T cell receptor that is responsive to riboflavin metabolites unable to be synthesized by animals but produced by diverse pathogenic and commensal GM organisms. These riboflavin metabolites are presented to MAIT cells by the major-histocompatibility related protein 1 (MR1), resulting in MAIT cell activation [51]. Loss of MR1 exacerbates GVHD in the gut in animal models [59]. Similarly, bacterial lipids which bind to CD1d on gut dendritic cells can potentiate NKT cell development and activation [49]. Butyrate-producing bacteria metabolizing host bile acids alter epigenomic acetylation of the FoxP3 locus leading to Treg development that can be co-opted to protect against autoimmune disease in GF mice; however, this also impairs anti-tumor responses [53,60]. Although absolute CD8^+^ T cell counts are not altered in GF environments, microbial-derived butyrate promotes memory T cell activation and survival by subverting glycolysis and directly promoting fatty acid use in oxidative phosphorylation [52].

Short chain fatty acids can drive innate immunity by activating human macrophage cell lines thereby promoting local gut immunity [46]. Pathogen-associated molecular patterns (PAMPs) for GM bacteria also directly stimulate the differentiation and growth of dendritic cells and potentiate protective immunity in non-GI organs [47]. In antibiotic-treated neonatal mice decolonized of commensal bacteria, stimulatory microbial signals to innate lymphoid cells (ILCs) are lacking, resulting in decreased IL-17 (IL-17) and granulocyte colony stimulating factor (G-CSF) production with subsequent impaired granulopoiesis and susceptibility to *Escherichia coli* sepsis [44]. Impaired ILC type 3 development in antibiotic exposed neonatal mice also directly impairs mucosal immunity and increases susceptibility to *Klebsiella pneumoniae* [61].

## ALTERATIONS IN THE GM FOLLOWING ALLO-HSCT

From species identification by 16S rRNA sequencing (16Sseq) of stool it is known that GM bacterial diversity in adult and pediatric patients undergoing allo-HSCT is less than that seen in healthy controls and may take up to five months to recover [62–64]. At a species-specific level, in children there is a shift away from microbes commonly associated with healthy GM, towards more pathogenic genera such as *Staphylococcus and Enterobacter*. Likewise, in adults a dysbiotic shift towards *Enteroccocus* within phylum *Firmicutes* and an increase in obligate anaerobes is also observed [62,64,65]. The reasons underlying these marked post-allo-HSCT changes in GM composition are complex and interacting. Exposure to antibiotics, radiation and chemotherapy prior to transplant can be directly toxic to some GM species, and conditioning therapy often disrupts mucosal barriers and impairs mucosal immunity [66,67]. Disruption of the intestinal barrier by radiation, conditioning chemotherapy or gut GVHD may lead to loss of Paneth cells with subsequent decrease secretion of defensins and overgrowth or normally defensin-sensitive species [68]. The loss of the intestinal mucosal barrier may also exacerbate GVHD and lead to a reliance on total parenteral nutrition which in turn is associated with dysbiosis [69].

Technological and statistical limitations may also limit a complete appreciation of the GM post-allo-HSCT and subsequent functional implications of dysbiosis. Clustering of 16Sseq data as operational taxonomic units (OTUs) allows classification and grouping of similar sequences as OTUs, but usually limits bacterial assignment to genera, thus eschewing species-specific effects. This potentially dilutes competing effects of species within the same genus; and often does not address the contribution of the virome and fungome [70,71]. Furthermore, bacteria exist in communities, share genetic elements, and display metabolic cooperativity that cannot be comprehensively captured by taxonomic identification [72]. New approaches, such as shotgun metagenomic sequencing (SMGS) allow identification of individual bacterial, viral and fungal species and allow associations at the gene-level, providing an opportunity for more complete and mechanistic associations of the GM with clinical outcomes yet are contingent on the ever-increasing annotation of microbial genomes [73,74]. Creating associations between GM and transplant outcomes from large datasets may also require the use of novel, iterative statistical approaches that are dependent on large-scale computing power and take into account prior probabilities, such as sequencing depth, as well as draw associations not only between total read count of genes but variations in reads [75,76]. Such Bayesian and frequentist methods are already being implemented as outlined in the subsequent section [7,8].

## ASSOCIATION OF THE GM WITH POST-ALLO-HSCT OUTCOMES AND IMMUNE RECONSTITUTION

### Empirical Evidence from Murine Model Systems

The influence of the GM on allo-HSCT outcomes is most clearly seen in antibiotic-treated SPF and gnotobiotic mouse models where the GM composition can be controlled [77]. Innate immune cells activated by gut dysbiosis through pathogen sensing TLRs produce reactive oxygen species that can mediate tissue injury in mouse GVHD [78]. Co-housing of transplanted wild-type (WT) mice with IL-17−/− mice susceptible to hyperacute GVHD, results in altered GM in WT mice and subsequent increased aGVHD susceptibility [79]. The post-allo-HSCT GM has also been shown to increase IL-12/23p40 subunit liberation that upregulates MHC-II on intestinal epithelial cells and initiates lethal GVHD [80]. Lactose excess has been shown to drive *Enterococcal* domination that is associated with aGVHD, whilst microbial liberation of indoles elicits a type 1 interferon signal, paradoxically shown to protect against aGVHD [67,81].

Despite the mechanistic associations of immune development with microbial metabolism in the gut and the marked alterations in gut microbiota following allo-HSCT, few reports have drawn direct links between the gut microbiome and post-allo-HSCT immune reconstitution in mouse models. Staffas at al. demonstrated that antibiotic-treated mice showed decreased granulocyte and lymphocyte recovery following allo-HSCT [6]. In a series of elegant experiments it was further shown that liberation of sucrose from gut microbiota post-allo-HSCT stimulates granulopoiesis and that sucrose supplementation can be used as a post-biotic to improve neutrophil recovery in antibiotic treated mice [6]. Previous studies have also demonstrated that GF and antibiotic-treated mice show reduced HSC and thus impaired normal granulopoiesis and lymphopoiesis [82,83]. However, the study by Staffas et al. showed that HSC numbers were relatively unaffected indicating that the GM may alter more differentiated progenitor cells after allo-HSCT compared to normal immune development [6]. This hypothesis comes with the caveat that these studies differed in the use of antibiotic-treated mice versus GF mice and the regimen of antibiotics used.

### Clinical Associations in Patients

In adult patients, associations between GM and allo-HSCT outcomes rely on temporal delineations between gut dysbiosis, immune reconstitution and transplant outcome or therapy-induced alterations in the GM that result in clinical outcomes. In the largest study to date, van den Brink and colleagues showed in a multi-center study comprising 1362 patients at four centers that microbial diversity in the peri-engraftment, but not pre-engraftment, period is associated with overall survival when controlling for antibiotic exposure [71]. Interestingly, the effect of stool microbial diversity on overall survival was only seen in the subset of patients who received T cell replete grafts implying an interaction between T cell immune reconstitution and the GM [71]. In a recent follow-up study from the same center, Bayesian regression was used to show decreased microbial diversity was associated with impaired neutrophil and lymphocyte recovery [8]. Corroborating results seen in mice undergoing allo-HSCT [6], it was further shown that Ruminococcus 2 species were associated improved lymphocyte recovery [78]. Further reports from this large cohort detailing exact immune subsets associated with recovery are awaited.

Other single center studies have shown an association between incidence and severity of aGVHD and subsequent mortality and reduced GM diversity in the peri-engraftment period with only one study documenting an association of GVHD with pre-transplant GM composition [64,84–90]. Confirming these results, antibiotic exposure both and pre- and post-allo-HSCT decreases GM diversity and increases the risk of GVHD [66,77,91]. Dysbiosis and bacterial predominance within the GM can also lead to translocation and subsequent blood stream infection with attendant morbidity and mortality, even with species not commonly associated with gut colonization such as *S. epidermidis* and *P. aeruginosa* [92]. The association of species loss and increased risk of GVHD is further corroborated by evidence of the utility of fecal microbiota transplantation (FMT) to increase GM diversity and thus treat even steroid-refractory acute GVHD whilst also enhancing monocyte and lymphocyte recovery [8,93–96].

In pediatric populations there is also evidence of the interaction between the post-allo-HSCT GM and transplant outcomes, yet with only one study to data reporting associations with immune reconstitution. Antibiotic exposure is associated with decreased commensal species of the Clostridial class and subsequent increased risk of gut GVHD in children post-allo-HSCT [97]. Other pediatric studies have confirmed results seen in adult patients showing an association of GVHD with a loss of *Blautia* spp. [71,98].

In a comprehensive, data-dense study, Ingham et al. undertook longitudinal monitoring of immune recovery, markers of inflammation and GM composition in 37 children prior to and following allo-HSCT and confirm that in children microbial diversity markedly decreases following allo-HSCT and did not return to pre-allo-HSCT levels after 5 weeks. Using multivariate regression analysis with sparse partial least squares regression (sPLS) followed by hierarchical clustering to identify explanatory variables and perform dimensionality reduction [99], it was shown that monocyte recovery correlated with microaerophilic *Lactobacillus* spp. in stool. Supporting this association, is empirical evidence that the pili of *Lactobacillus* spp. known to thrive in microaerophilic conditions in the gut, such as *Lactobacillus rhamnosus* GG, stimulate monocytes via TLR2 [100]. Using another iterative technique that facilitates the integration of categorical and continuous clinical data [75], canonical correspondence analysis (CCpnA), Ingham et al. derived associations between the GM, immune recovery and GVHD post-allo-HSCT. CCpnA clustering was similar to that observed with sPLS, and revealed an association between severe GVHD, high monocyte counts and *Lactobacillus* spp. [7]. It would be interesting to further define M1/M2 polarization of macrophage recovery in this study, given that orally administered *Lactobacillus* spp. have been shown to drive M1 polarization in murine bone marrow derived macrophages [101] and M1 polarization has also been associated with GVHD in mice and humans [102,103]. Assessing adaptive immune responses, Ingham et al. also showed that early NK and B cell reconstitution were associated with less severe GVHD and a predominance of obligate anaerobes of the clostridial order *Ruminococcacae* and *Lachnospiracae* in the GM [7]. B cell expansion can be stimulated by superantigens from these species; however, the precise role of B cells in GVHD is more complex—early naïve B cell recovery may be associated with less severe GVHD, whilst continued B cell secretion of autoantibodies may drive chronic GVHD in the later months post-allo-HSCT [29]. Further studies assessing the precise immunophenotype and transcriptome of these cells may elucidate the exact immune subsets and potential mechanisms driving these associations in this pediatric cohort.

The mechanisms by which alterations in the GM impact immune reconstitution in humans is mostly speculative. Presence of microbial-derived short chain fatty acids, such as butyrate, in serum of patients have recently been shown to be inversely associated with chronic GVHD; however, the effect of butyrate on Treg reconstitution in thus study was not shown [104]. We and others have shown bacteria capable of producing riboflavin metabolites are associated with MAIT cell recovery and decreased severity of GVHD, supporting existing empirical observations in mouse models [34,59,105]. Using PICRUSt—a bioinformatic tool to predict metabolic pathways present in organisms sequenced by 16Sseq [106]—Konuma et al. showed that organisms that would be predicted to express the riboflavin metabolizing genes, *ribA and ribB,* were associated with MAIT cell recovery. However, without gene-level evidence of involvement using SMGS, such functional effect is unable to be confirmed.

## CONCLUSIONS AND FUTURE DIRECTIONS

There is a large and growing body of evidence linking GM composition with normal immune development. Similar associations have been reported in interactions between the GM and post-allo-HSCT immune reconstitution and outcomes, yet gaps remain. Identification of immune subsets that are affected by distinct microbial species may guide directed FMT, pre-biotic and post-biotic therapies post-allo-HSCT. In addition to new technologies such as SMGS and iterative statistical methods that take advantage of increasing computational power will be able to identify associations in large, multi-table microbial and clinical datasets in relatively small groups of patients without being subject to increased identification of false positives [75]. Utilizing more robust identification of species- and gene-specific associations with immunophenotypes, transcriptomes and clinical outcomes and correlating these data with findings in normal immune development may lead to directed manipulations of the GM and/or metabolic pathways that will improve immune reconstitution and outcomes following allo-HSCT.

## Figures and Tables

**Table 1.** Association of immune cell subsets with relapse, infection and acute graft versus host disease from patient data.

Immune cell subset	Effect on GVM and infection [refs]	Effect on acute GVHD [refs]
**Neutrophil**	Early recovery has no clear effect on GVM. Protects against opportunistic infection [30]	Early recovery associated with increased risk of GVHD [30]
**Monocyte**	Early recovery associated with less relapse in certain subsets [31]	Timing of recovery not clearly associated with aGVHD [15]
**Dendritic cell**	Low plasmacytoid dendritic cell recovery associated with relapse and infection [32]	High plasmacytoid and myeloid dendritic cell recovery associated with less incidence of severe aGVHD [32,33]
**NK cell**	Early recovery associated with less relapse [13,14]	Early recovery associated with increased risk of aGVHD [13,14]
**NKT Cell**	Early recovery associated with less relapse [3,24,29]	Early recovery associated with increased risk of aGVHD [3,24,29]
**γδ T cell**	Early recovery associated with less relapse and infection [26–28]	No Effect on aGVHD [28]
**MAIT cell**	No clear association with infection or relapse [3]	Early recovery associated with less GVHD [25,34]
**CD8^+^ T cell**	Early recovery associated with less relapse and infection [19–22]	Early recovery associated with increased risk of aGVHD [19–22]
**CD4^+^ Th cell**	Subset dependent. Mostly associated with decreased risk of infection and relapse. [3,21]	Subset dependent. Mostly associated with increased risk of aGVHD [3]
**CD4^+^ Treg cell**	Early recovery may be associated with increased risk of relapse [33]	Higher cell numbers associated with less aGVHD [3]
**B cell**	Early recovery associated with less infection [14,29]	Early recovery associated with decreased risk of aGVHD [29]

Abbreviations: aGVHD, acute graft versus host disease; NK, natural killer; Th, T helper cell; Treg, T regulatory cell.

**Table 2.** Examples of the impact of gut microbiota on immune cell development in mice and human in vitro model systems.

Immune cell	Model system [ref]	Bacteria	Bacterial component & mechanism
**Neutrophil**	Antibiotic-treated mice [44]	Loss of: Proteobacteria (early); Firmicutes, class: Bacilli (day 5–14); Bacteroidetes, class: Bacteroidia (late)	↓ PAMP → ↓ stimulation of ILC → ↓ GCSF → neutrophils
	Antibiotic-treated allo-HSCT mice [6]	↓ bacterial load and diversity	↓ bacterial metabolism → ↓ digested sugars → ↓ neutrophils
**Monocyte**	Antibiotic-treated mice [45]	Loss of *Lactobacillus*	↓ SCFA → ↓ monocyte activation
	Human U937 monoblastic leukemia cell line [46]	SCFA producing bacteria (phyla Firmicutes and Bacteroidetes)	↓ SCFA → ↓ inflammasome complex → ↓ monocyte activation
	Stimulated human healthy donor cells [43]	*Streptococcus pyogenes*	↓ exotoxin → ↓ monocyte stimulation and differentiation
**Dendritic cell**	Gnotobiotic GF and comparison with SPF mice [38]	*Bacteroides fragilis*	Polysaccharide → ↑ TLR2 activation → plasmacytoid DC development
	Antibiotic-treated mice [47]	*Lactobacillus plantarum*	Glycolipid → mincle receptor on DC → DC activation
**NK cell**	Lactobacillus treated healthy human donor cells [48]	*Bifidobacterium bifidum* and *Lactobacillus reuteri*	Stimulation of DC → activation and expansion of NK cells
**NKT Cell**	Gnotobiotic GF mice [49]	*Sphingomonas* spp.	Bacterial lipids → bind to CD1d → NKT stimulation and expansion
	Comparison of GF and SPF mice [39]	*Bacteroides fragilis*	Polysaccharide → ↑ TLR2 activation → DC migration from gut to thymus → thymic iNKT development
**MAIT cell**	Comparison of GF and SPF mice [50]	Riboflavin metabolizing bacteria (e.g., *Blautia* spp.)	5-ARU → 5-OPRU → bound to MR1 → MAIT cell stimulation and expansion
	Healthy human donor cells stimulated by MR1 bound riboflavin metabolites [51]	(No bacteria used)	MR1 bound riboflavin metabolites → MAIT cell stimulation and expansion
**CD8^+^ T cell**	Comparison of GF and SPF mice [52]	Specific bacteria not identified	Microbial-derived butyrate → ↓ glycolysis and ↑ oxidative phosphorylation → promotes memory formation from activated T cells
**CD4^+^ Th cell**	Gnotobiotic GF and comparison with SPF mice [38]	*Bacteroides fragilis*	Polysaccharide → ↑ TLR2 activation → plasmacytoid DC development → ↑ Th cell
	Treatment of SPF mice with bacteria	*Bacteroides fragilis*	Polysaccharide → binds to B cells → CD4^+^ Th development
	Gnotobiotic GF	Segmented filamentous bacteria	Adherence to mucosa → SAA and ROS → TH17 development
**CD4^+^ Treg cell**	Gnotobiotic GF mice [53]	*Clostridioides difficile*	Butyrate production → epigenomic change at FoxP3 locus → ↑ Treg development
**B cell**	Gnotobiotic GF mice [54,55]	*Escherichia coli* K12 variant	High bacterial load in gut → ↑ B cell IgA repertoire

Abbreviations: PAMP, pathogen association molecular pattern; GCSF, granulocyte colony stimulating factor; allo-HSCT, allogeneic hematopoietic stem cell transplantation; SCFA, short chain fatty acid; GF, germ free; SPF, specific-pathogen-free; TLR, toll-like receptor; DC, dendritic cell; NK, natural killer; 5-ARU, 5-amino-6-d-ribitylaminouracil; 5-OPRU, 5-(2-oxopropylideneamino)-6-d-ribitylaminouracil; MAIT, mucosal-associated invariant T cell; Th, T helper cell; Treg, T regulatory cell; SFB, segmented filamentous bacteria.
